# Stridor in an Elderly Woman: An Unusual Presentation of a Giant Thyroglossal Cyst

**DOI:** 10.1155/2013/340814

**Published:** 2013-08-13

**Authors:** Sithananda Kumar Venkatesan, Kiruba Shankar Manoharan, Pradipta Kumar Parida, Arun Alexander, S. Gopalakrishnan

**Affiliations:** Deptartement of ENT and Head & Neck Surgery, JIPMER, Puducherry 605006, India

## Abstract

Thyroglossal cysts are one of the most common midline neck masses. They usually present as midline painless cystic neck mass in the first three decades of life. These anomalies are very rare in elderly patients and may pose difficult diagnostic and therapeutic challenges. Here, we report a case of giant thyroglossal cyst in a 72-year-female patient who presented with stridor, hoarseness of voice, and vocal cord paresis with gross distortion of normal airway anatomy secondary to pressure effect of the mass. The gross distortion and displacement of airway along with respiratory distress in this patient posed a difficult situation in securing the airway. The airway was secured by a unique way of orotracheal intubation with the help of a ventilating airway exchange catheter. The cyst was excised in toto under general anaesthesia. The stridor completely resolved after surgery and tracheostomy was avoided.

## 1. Introduction

Thyroglossal cysts are one of the most common nonneoplastic neck masses in the paediatric population [1]. Thyroglossal cysts are formed due to aberrations in the normal development and descent of the median thyroid anlage. The thyroglossal duct begins to degenerate between the fifth and sixth fetal weeks [2]. Failure of involution of this tract with secretory activity of the lining epithelial cells secondary to infections leads to the formation of a thyroglossal cyst [2]. It is rare in the elderly patients with a reported incidence of approximately 0.6% in the 6th decade [3]. Thirteen cases have been reported in the literature after the age of 70 [3]. It usually presents as a painless midline neck mass intimately related to the hyoid bone and moves with protrusion of tongue and deglutition [1]. Patients with thyroglossal cyst may present with unusual symptoms like dysphagia, respiratory distress, and discharging fistulas.

## 2. Case Report

A 72-year-old female patient presented to the emergency otolaryngology services with a huge anterior neck swelling, hoarseness of voice, and stridor. The swelling started as a painless lump in the upper part of neck which gradually increased in size over 5 years. The patient developed progressive difficulty in breathing and hoarseness of voice of one-month duration and noisy breathing (stridor) of 4-days-duration which brought her to the emergency department. There was no history of recent rapid increase in size of the swelling. The patient did not give history suggestive of any neurological illness, prior surgery, or trauma.

**Figure 1:**
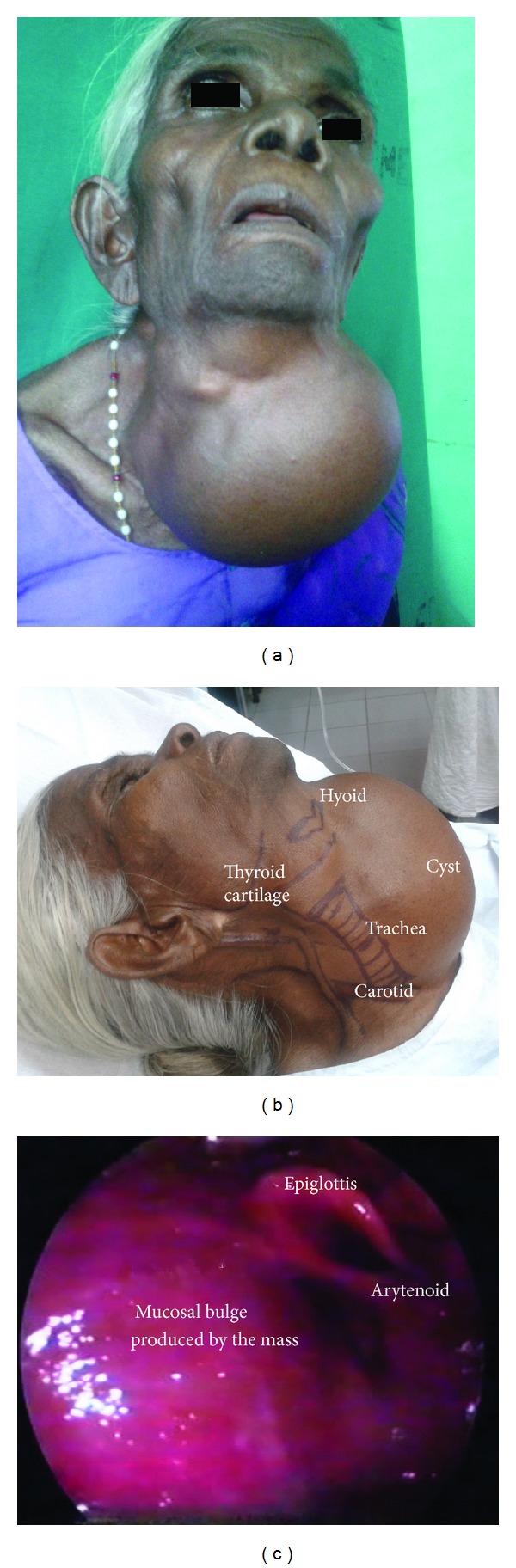
(a) Patient with neck mass. (b) Surface marking of neck structures displaced and distorted by the cyst. (c) Endolaryngeal picture showing airway distortion.

On examination, the patient had severe stridor and hoarseness of voice which worsened in supine position. The pulse rate was 110 per min and regular in rhythm. The blood pressure was 110/80 mm of Hg. The respiratory rate was 42 per minute. Neck examination revealed a 20 cm × 18 cm mass in the anterior neck more on the left side extending from the level of hyoid superiorly to the level of the suprasternal notch inferiorly (Figure 1(a)). The mass extended from the posterior border of left sternocleidomastoid to the anterior border of right sternocleidomastoid crossing the midline. The mass moved with deglutition but not with protrusion of tongue. The hyoid, laryngeal framework, and the trachea were pushed towards the right side (Figure 1(b)). The right carotid pulse could be felt along the lateral border of the mass (Figure 1(b)). Laryngoscopy with 70 degree 4 mm rigid endoscope (Karl Storz) revealed a distorted upper airway with folded epiglottis and a narrowed glottis pushed to the right (Figure 1(c)). The right cord was mobile and the left cord was paralysed. Based on history and clinical examination a provisional diagnosis of a malignant lesion of thyroid was made with a differential diagnosis of cystic colloid goitre and branchial cyst.

**Figure 2:**
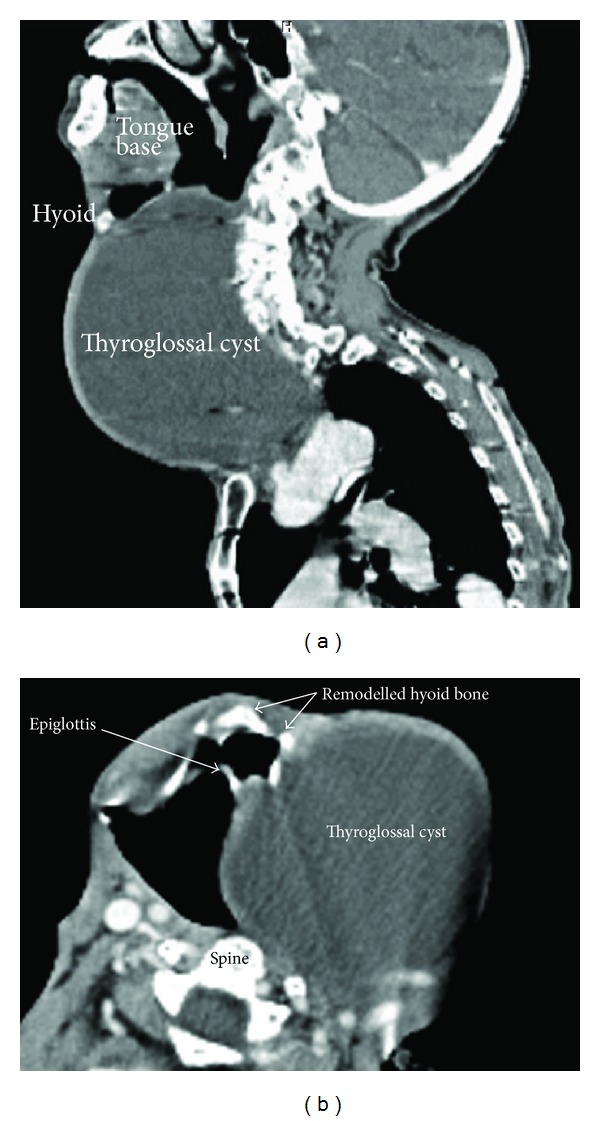
(a) C. T. scan sagittal section showing the relationship of the cyst with hyoid bone and airway. (b) C. T. scan axial section showing the remodelling of hyoid bone in relation to the cyst.

Ultrasound revealed a 14 cm cystic mass which was hypoechoic with homogenous echogenicity compressing the airway. The right carotid was found to be pushed laterally. Contrast enhanced computed tomography (CECT) showed a homogenous mass of size 14 × 10 cm on the left side extending from the hyoid to the manubrium sterni compressing the tracheal lumen to a longitudinal slit. The cyst was intimately related to the hyoid bone. There was distortion of the laryngeal framework with localized remodelling of hyoid bone (Figures 2(a) and 2(b)). Baseline haematological and biochemical parameters and chest radiograph were normal.

As the airway anatomy was distorted, difficult intubation was anticipated. The airway was secured in the awake patient, with endoscopic guidance by railroading the endotracheal tube over number 14.0fr/65 cm ventilating airway exchange catheter (FROVA, COOK INCORPORATED, USA). Intraoperatively approximately 14 cm × 10 cm cystic mass was seen compressing the left lobe of thyroid gland. The left recurrent laryngeal nerve was found to be displaced from the tracheo-esophageal groove and stretched beneath the mass.

The left lamina of thyroid cartilage, the cricoid cartilage, and the tracheal rings were distorted and compressed by the mass. The cyst was partially decompressed by needle aspiration which helped to identify the tissue planes and the entire cyst was removed completely after identifying the recurrent laryngeal nerve on both sides. The patient was extubated after surgery without any need for tracheostomy. 

Histopathological examination revealed a cyst with squamous epithelial lining with underlying thyroid tissue. There was no evidence of malignant foci within the cyst. The findings were consistent with a benign thyroglossal cyst. The cyst fluid was sterile with plenty of exfoliated epithelial cells and negative for malignant cytology. The postoperative period was uneventful. Post-op thyroid function tests revealed an euthyroid status. The stridor resolved completely immediately after surgery but hoarseness persisted. The patient was discharged on the 10th post-op day. No recurrence was found in the patient on followup three months after surgery.

## 3. Discussion

In this case report we want to highlight the atypical presentation with acute airway compromise in an elderly patient with thyroglossal cyst and the novel approach of securing the distorted airway by avoiding tracheostomy and traumatic intubation. This report is intended to communicate our experience in managing a case of thyroglossal cyst in a 72-year-old lady which has reached giant proportions (14 cm × 10 cm) and presented with severe stridor, hoarseness of voice, gross distortion of airway anatomy, and vocal cord paralysis secondary to mass effect of the cyst. To our knowledge there has been no report of thyroglossal cysts presenting with acute airway compromise and vocal cord palsy in the elderly patients in the English literature [2–7]. This atypical acute presentation with extensively distorted airway anatomy and the possibility of tracheomalacia due to longstanding tracheal compression precluded us from securing the airway by emergency tracheostomy or blind oro-tracheal intubation. The airway was secured in the awake patient under fibre-optic guidance with ventilating airway exchange catheter.

The recommended treatment of thyroglossal cyst in the elderly population varies and includes medical therapy, no intervention, and surgical treatment [4]. A classical Sistrunk operation could not be contemplated in this patient because of acute and atypical clinical presentation, lack of preoperative tissue diagnosis, and the possibility of hyoid excision jeopardising the already distorted and compromised airway. Preoperative aspiration of the giant cystic swelling with distorted neck anatomy helped in identification of tissue planes and preservation of vital structures. 

## 4. Conclusion

Thyroglossal cyst should be considered in the differential diagnosis of a large neck mass presenting with stridor and cord palsy in the elderly. In case of gross distortion of airway, as in this case, traumatic intubation and tracheostomy can be avoided by using a ventilatory airway exchange catheter. Controlled decompression of the cyst by intraoperative aspiration helps in identifying and preserving vital structures like the recurrent laryngeal nerve.
